# Cross-talk between microbiota and immune fitness to steer and control response to anti PD-1/PDL-1 treatment

**DOI:** 10.18632/oncotarget.12985

**Published:** 2016-10-28

**Authors:** Andrea Botticelli, Ilaria Zizzari, Federica Mazzuca, Paolo Antonio Ascierto, Lorenza Putignani, Luca Marchetti, Chiara Napoletano, Marianna Nuti, Paolo Marchetti

**Affiliations:** ^1^ Department of Experimental Medicine, Sapienza University of Rome, Rome, Italy; ^2^ Department of Clinical and Molecular Medicine, SantAndrea Hospital, Sapienza University of Rome, Rome, Italy; ^3^ Units of Parasitology and Human Microbiome, Bambino Ges Childrens Hospital and Research Institute, Rome, Italy; ^4^ Melanoma, Cancer Immunotherapy, and Innovative Therapy, Istituto nazionale Tumori Fondazione G Pascale, Napoli, Italy; ^5^ Department of Clinical Oncology, Policlinico Umberto I, University of Rome Sapienza, Rome, Italy

**Keywords:** microbiome, immunotherapy, PD-1, PDL-1, CTLA-4

## Abstract

Immune Checkpoint Inhibitors (ICIs) are improving the survival of cancer patients, however only the 20-30% of treated patients present clinical benefits. Toxicity represents the major cause of reduced dosage, delayed drug administration and therapy discontinuation. Hence in the context of multiple treatment possibilities, the identification of predictive markers of response and toxicity is a challenging approach for drug selection in order to obtain the best clinical benefit while minimizing the side effects. The loss of the protective function of intestinal barriers that interacts with the environment measured as increased intestinal permeability and the changes occurring in the microbiota composition have been proposed as a mechanism potentially explaining the pathogenesis of immune related toxicity.

In this review we discuss the new perspectives on the involvement of PD-1 and PDL-1 in the cross talk between gut microbiota and immune fitness and how gut microbiota impacts on the efficacy of anti-PD-1 and anti-PDL-1 treatments in cancer.

## INTRODUCTION

In the last years the modulation of immune checkpoint network is becoming an important therapeutic strategy for anti-cancer treatments. The activation of immune system able to kill the tumor represents the goal of cancer immunotherapy [1].

In anti-tumor immune response T lymphocytes represent the major components. The optimal recognition of the antigen induces a specific activation of T cells, followed by the acquisition of the effector function. It is particularly significant the differentiation of a specific subset of T cells, the cytotoxic T lymphocytes (CTLs), that are able to lyse target cells. In cancer, such as in chronic viral infection, the long exposure to the antigen leads to a dysfunction of T cells; in particular these cells lose their proliferation ability and progressively the capability to release cytokines, to eliminate pathogens and to kill target cells. This condition represents the state of “exhaustion”. Recent findings have defined the function of some receptors that negatively regulate T cell activity and promote exhaustion[2].

The intuition that targeting these receptors could dramatically influence T cell activity was originally of James P. Allison in his pioneer studies on cytotoxic T-lymphocyte antigen 4 (CTLA-4) inhibitory receptors. The idea was that if the negative regulation of T cells could be blocked, T cell responses would be expanded and sustained long enough to eliminate cancer [3][4]. The other important insight was translating the target from the cancer cell and its antigenic/genomic repertoire to the immune system unregarding the type of tumor and the antigens expressed. Several antibodies to different immune checkpoint inhibitors (ICIs) where then generated and tested in preclinical setting. They demonstrate capacity to unleash existing T cells in a unspecific mode, thus breaking the tolerance against self and non self neoantigens associated with the tumor and permitting the expansion of effector T cells able not only to recognize but also to destroy the tumor. Initial clinical trial results were exciting, ICI therapy led to tumor regression and improved survival in a subgroup of metastatic melanoma, lung carcinoma, renal cell carcinoma and lymphoma patients. Clinical trials are currently exploring combination therapies. The first ICIs approved by FDA are directed against the CTLA-4, programmed death receptor-1 (PD-1) and programmed death receptor-1 ligand (PD-L1) [5] .

A distinguishing feature that was observed since the first report of ICI anti-CTLA-4 treatment in metastatic melanoma patients is the observation that the responding patients showed durable complete responses. The response is maintained for a long time after the end of the treatment and long time survivors up to ten years and cured patients are now a reality [6] .

Several factors appear to governate the efficacy of these treatments. Pre existing endogenous natural or induced anti tumor immunity is one of the variables that has been associated with increased response. Interference with inhibitory pathways in the effector T cells and concomitant removal of immune-suppressive cells such as Treg cells are also dominant mechanisms of enhanced anti-tumor activity [7]. Oncologists have now tested the powerful potential of ICI treatment in cancer. Activated T cells unleashed from negative brakes are able to rapidly find target tumor cells, kill also significant tumor burden and maintain memory and control of recurrences. In order to proceed with novel combination of ICI and integration of these novel treatments with chemo/radiotherapy and target therapies, oncologists are now focalizing attention and research efforts on the management of novel array of immune related toxicities. The new side effects described for ICI treatments are in fact mainly immune related and autoimmunity classified, distinct from chemo and molecular targeted therapy and they have challenged greatly medical oncologists [8] . The most common toxicity observed included: diarrohea, colitis, thyroid disfunction, hypofisitis, liver disorder, dermatologic event and lung disorder. Altough these immune related side effects have become maneageable to some extent by the use of corticosteroid therapy, new predictive indicators of response and toxicity are necessary to improve the management and the compliance to immunotherapy.

In this setting among all the fields that are being explored, the study of the microbiome is showing interesting results mainly for two reasons, one comes from recent studies that have addressed the critical role that microbiome appears to have in the development of inflammation, cancer and in the integrity of mucosal immunity and the protection against pathogens. Second the high frequency of the severe diarrhea and colitis affecting ICI treated patients confirming a role of gut microbiome and suggesting possible microbiota influence on the therapeutic activity/toxicity of ICI immunotherapy.

In this review we discuss the new perspectives on the involvement of PD-1 and PDL-1 in the cross talk between gut microbiota and immune fitness and how gut microbiota impacts on the efficacy of anti-PD-1 and anti-PD-L1 treatments in cancer.

## PD-1/PD-L1 AXIS: IMMUNOLOGICAL AND CLINICAL IMPLICATIONS

PD-1 receptor, also called CD279, represents one of the most important target for immunological therapy. It is an inhibitory receptor expressed by activated T lymphocytes, B cells, natural killer T cells (NKT) and Treg cells [9]. PD-1 is a member of the CD28 co-receptor family [10] and has a key role in the modulation of T cell function in peripheral tissue, recognizing PD-L1 and PD-L2. Both these ligands are expressed on antigen presenting cells (APCs); in addition PD-L1 is present also on the surface of several cells of lymphoid and non lymphoid tissue and it is expressed by tumor cells [11]. The function of PD-1 is mainly regulated by its cytoplasmatic domain, containing an immunoreceptor tyrosine-based inhibitory motif (ITIM) and an immunoreceptor tyrosine-based switch motif (ITSM). When PD-1 recognizes its ligand, this interaction induces the phosphorylation of the tyrosine residue in ITSM, recruiting the tyrosine phosphatase SHP2 that induces the dephosphorylation and inactivation of Zap70 in T cells, down regulating TCR signaling activation. Therefore PD-1 down regulating T cell activity, affects negatively immune response. When in tumor microenvironment PD-1 binds PD-L1, T cell function is attenuated, so that T lymphocytes become unable to target tumor cells. Hence anti-tumor response results strongly restrained and tumor evasion favored. Initially the role of PD-1 in modulating T cell activity was described in chronic viral infection. It was shown that during chronic infection of LCMV all specific CD8^+^ T cells expressed PD-1, instead during acute infection this receptor has not been detected on LCMV-specific memory CD8^+^ T cells. [12]. Since the interaction between PD-1/PD-L1 can be blocked by monoclonal antibodies, these are now considered novel therapeutic approaches to unleash the anti-tumor immune response. In fact it has been strongly suggested that immune evasion of cancer can be favored by the expression of PD-1 by tumor infiltrating lymphocytes (TILs) along with the expression of PD-L1 by tumor cells [13]. Many studies have shown that blockade of PD-1 or PD-L1 restores T cell function, induce an increase of IFNγ [14] and a decrease of immune suppressive cell subsets, such as MDSCs [15]. In fact PD-1 and PD-L1 blocking represents an extremely efficient approach in controlling tumor growth by changing the dynamic of the tumor microenvironment. Currently different monoclonal anti PD-1 and anti-PD-L1 antibodies are in development for the treatment of advanced disease; they include Nivolumab (OPDIVO, anti-PD 1) [16–23], Pembrolizumab (Keytruda, anti-PD-1) [24–34], Atezolizumab (anti PD-L1)[35–36] , Durvalumab (anti-PDL-1)[37–38] and many others. These agents while are revolutionizing cancer patients care[39] , have a precise pattern of toxicity, that can be classified as immune related. It is important today to understand better the variability observed in patient outcomes together with strategies to improve efficacy and identify parameters to select responsive patients. Microbiota could represent one physiological mechanism that can influence and modulate response to ICI treatments. The involvement of gut microbiota in the outcome of anti cancer therapy and the role of immune response create new questions from a preclinical and clinical standpoint in the cancer field [40] .

## MICROBIOME AND CANCER

Gut microbiota complexity and behaviour deserve the definition of *tissue organ*, as introduced and thoroughly discussed by Burcelin and collaborators [41], a major immunological organ which means metabolic organ, that influences different pathways of whole metabolism.Therefore the intricacy of microbiota components, metabolic functions and signaling control of the host leads to revise the concept of gut-host relationship in term of gut-microbiota-host network. In particular there is a close relationship between the acquisition of microbiome and the maturation of immune system during ontogeny. Intestinal homeostasis is then maintained through an efficient and interacting immune network that permits tolerance to the microbiota while allowing responsiveness to invading pathogens. Different members of the microbiota and their components have been demonstrated to interact with specific immune components influencing the synthesis of regulatory cytokines.

The final decision towards tolerance vs reactivity is the result of integrated signals from the microbiota and immune/non immune cells in the local microenvironment [42] . The perturbation of gut microbiota, called intestinal dysbiosis, is involved in many pathological mechanisms. Recent studies demonstrated the associations between microbiota profiles and the development of adiposity, diabetes, dyslipidemia and other inflammatory conditions.[43–48]

The close association between cancer susceptibility [49–61], responsiveness to cancer therapy and microbiome has just been investigated. Infact it was shown that the production of IL-17 in response to change of microbiota composition is associated to rapid progression of colo-rectal cancer. Furthermore enteric bacterial genes metabolizing estrogens could modify the risk to develop hormone positive breast cancer in postmenopausal women. [62]

Recentely it was demonstrated that cyclophosphamide changes composition of microbiota and induces traslocation of bacteria (L*actobacillus jonsoniii and Enterococco hirae*) in secondary lymphoids organ, like spleen and mesenteric lymph nodes, stimulating the production of Th17 and Th1 cells [63], demonstrating that bacteria modulate chemotherapeutic drug efficacy. Furthermore in tumor bearing mice the perturbation of intestinal microbiota caused by antibiotics treatment is associated with the reduction of synthesis of cytokines and the decreasing effect of both CPG- oligonucleotides immunotherapy and chemotherapy. It was demonstrated that microbiome is also with inflammation modyfing the expression of gene involved.[64]. In this study the authors show that different microbiota profiles are associated with the TNF response. In particular the presence of *Ruminococcus* ( Gram negative), and *Alistipes* ( Gram-positive) is involved in TNF production , while an enriched Lactobacillus microbiota correlates with the fail of response.

Thus microbiota may have a crucial role in influencing cancer treatment efficacy and considering the close interaction with immune system it's reasonable to supposed its influence in response to ICIs or other immunotherapies.

In fact recently Vetizou et al. [65] demonstrated that germ free or antiobiotics treated mice had poor benefit from anti-CTLA-4 therapy and showed also that anti-CTLA-4 therapy can modify the composition of microbiota. Moreover a recent study established that microbiota composition enriched in *Bacteroides phlilym* can prevent the onset of immune colitis in patients treated with anti-CTLA-4 (Ipilimumab) [66]. This data support the idea that microbiota modifying immune response could influence the response of both chemotherapy and immunotherapy (Table 1).[67–72] Furthermore the microbiota profiles already studied in IBD and liver diseases could be useful to stratify cancer patients treated with ICIs [73–80].

**Table 1:** The immunological effects of gut microbiota

Bacteria	Model	Effects on immune system	
Lactobacillus johnsonii	mouse	Stimulates the differentiation of TH17 cells and Th1 cells	Viaud 2013
Enterococcus hirae	mouse	Stimulates the differentiation of TH17 cells and Th1 cells	Viaud 2013
Ruminococcus	mouse	TNF production, promotes response to immunotherapy	Iida 2013
Alistipes shahii	mouse	TNF production, promotes response to immunotherapy	Iida 2013
Lactobacillus fermentuum	mouse	TNF production , impairs response to immunotherapy	Iida 2013
Bacteroides fragilis	mouse	Induces TH 1 in tumor draining lymph nodes. Promotes the maturation of intratumoral dendritic cells Increases the activity of anti-CTLA4 in vivo Reduces the inflammatory response Reduced histopathology signs of colitis induced by CTLA4 blockade	Vetizou 2015
Bacteroides thetaiotamicron	mouse	Increseas the activity of anti-CTLA4 in vivo Reduced the inflammatory response	Vetizou 2015
Bacteroidales	mouse	Decreased after CTLA4 blockade	Vetizou 2015
Burkholderiales	mouse	Decreased after CTLA4 blockade	Vetizou 2015
Clostridiales	mouse	Increased after CTLA4 blockade	Vetizou 2015
Bifidobacterium breve, Bifidobacterium longum, Bifidobacterium adolescentis	mouse	Enhanced dendritic cells activation Increased CD8 +T cell accumulation,	Sivan 2015
Bifidobacterium breve Bifidobacterium longu,	mouse	Improved the response to PDL-1 Improved IFNy levels	Sivan 2015
Bacteroidetes	human	Enriched in colitis-resistant patients treated with ipilimumab	Dubin 2015
Clostridium species	mouse	Stimulates the induction of suppressive FOXp3+ Treg	Geuking 2011
Bacteroides fragilis	mouse	Stimulates the induction of suppressive FOXp3+ Treg	Geuking 2011
Staphylococcus aureus	mouse	Converts CD4+ T cells into Foxp3+ Treg cell	Hardis rabe 2013
Bacteroidaceae	mouse	Decreases in mice PD-1−/−	Kawamoto 2012
Bifidobacterium	mouse	Decreases in mice PD-1−/−	Kawamoto 2012
Enterobacteriaceae	mouse	Increases in mice PD1−/−	Kawamoto 2012
Erysipelotrichaceae Prevvotellaceae Alcaligenacee TM7 incerte saedis	mouse	Increase in mice PD1−/−	Kawamoto 2012

## MICROBIOME and PD1-PD-L1 axis

It's known that PD1-PDL1 axis plays a key role in the regulation of immune system and that immunotherapy is more efficient in T cell inflamed tumors rather than in T cell deficient tumors. Recent data support the hypothesis that microbiota shapes innate and adaptive immune system influencing PD-1-PD-L1 axis. In particular Sivan et al compared melanoma growth in mice derived from two different mouse facilities (JAX and TAC) harboring different intestinal microbiota but genetically similar [81]. They observed an higher rate of melanoma growth in TAC mice and a better response to PD-L1 treatment in JAX mice. Moreover the investigated the relationship between microbiota and immune cells demonstrating that *Bifidobaterium* seems to positively influence the number of activated antingen-presenting cells. Moreover the administration of *Bifidobacterium* to TAC mice improves tumor control and IFNγ production. Surprisingly the authors demonstrated that the combination of modulation of microbiota with anti-PD-L1 antibody improved tumor control. These data are very exciting because strongly suggest that different species can activate or conversely inhibit immune response.

Moreover the microbiota influences the development of regulatory T cells in mice, in particular germ free mice showed a lower amount of suppressive Foxp3^+^ Treg cells in the gut and the colonization of *Clostridium* species or *Bacteroides fragilis* stimulates the induction of suppressive Foxp3+ Treg cells in the intestine of these mice [82]. Furthermore neonatal human CD4^+^ T cells can be converted into Foxp3^+^ Treg cells by *Staphylococcus aureus*.

In factS.au*reus* increases the expression of PD-L1 on APCs, and this is linked to the APCs ability to induce Foxp3+ Tregs. The interaction between PD-L1 and PD-1, expressed on T cells, prevents the TCR signaling within T cells, which leads to differentiation into Foxp3^+^ Tregs.

These data demonstrate a significant role of specific gut bacteria in influencing immune system and response to cancer therapies. But it's equally true that the gut microbiota is itself modulated by immune response. In fact intestinal microbiota plays a crucial role in the development of gut immune system representing one of the first barrier against pathogens. Germ free mice presented reduced Pejer's patches, levels of immunoglobulin A, intraepithelial lymphocytes and production of antimicrobial peptide. It was also demonstrated that recolonization with healthy mouse commensal microbiota can correct the immune deficiency.

Fargarsan showed that PD-1^−/−^ mice have a significant alteration in microbiota composition (reduction of anaerobic bacteria, of *Bifidobacterium* and *Bacteroidaceae*, increase in *Enterobacteriaceae* and at the general level, increase in members of the *Erysipelotrichaceae, Prevotellaceae, Alcaligenaceae* and *TM7 genera incertae sedis*) and it's supposed to be caused by a decreased capacity of IgA of binding bacteria[83]. Thus PD-1 is strongly associated with the maturation of antibody to maintain the integrity of intestinal mucosal barrier [84] .

One accredited hypothesis, proposed by Rescigno speculates that the immune system can be manipulated to alter gut microbiota composition. In this way microbiota could be induced to be less pro-inflammatory (i.e. more diverse and with a reduced level of innate immune activators), thus reducing susceptibility to inflammation or minimizing the progression of the damage [85–90].

## FUTURE DIRECTION OF IMMUNOTHERAPEUTIC APPROACH

**Figure 1:**
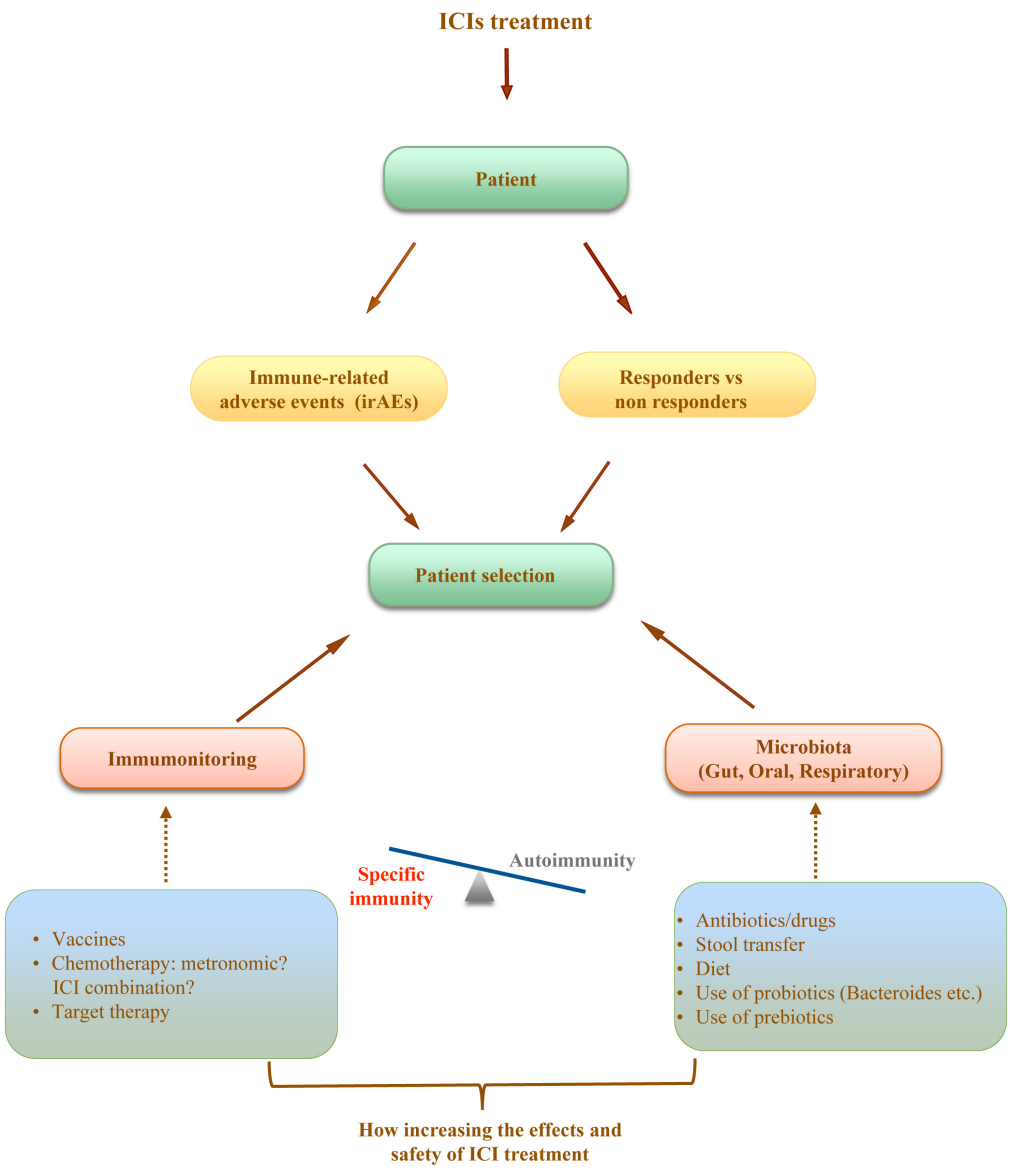
A new proprosed approach for the management of immunotherapy cancer treatment

The main challenge today for the oncologists is to fully utilize the potential of ICI treatment in order to treat and *cure* the majority of patients, to limit the immune related events and toxicity and to better understand the dynamics of response to treatment. It is conceivable that in a very short time ICI treatment will be proposed for all tumors and in earlier setting in the different protocols. In this review we outline several recent findings that could help to draw a roadmap of clinical and laboratory criteria to help the oncologist in designing more efficient protocols of ICIs treatment (Figure 1). We hypothesize that the identification of different microbiome profiles (for example *Bifidus* enriched or *Bacterioides* enriched) could help us to establish classes of patients responders or at major risk to develop high grade toxicities. To better define the profile of our patients we could also consider the nutritional status and immune repertoire. The possibility of intervention is attractive. In fact diet, use of probiotics, prebiotics and antibiotics or stool transfer that can change microbiota profile, drugs that can modulate mucosal permeability and homeostasis as well as pretreatment immunotherapy/chemotherapy to increase the specific anti tumor T cell compartment are some of the strategies. We are today dealing with oncology treatments that have moved the attention from the tumor to the patients immune system and the multiple intersecting immunity regulatory networks. The further understanding of these mechanisms and the relation with clinical outcome will be the key for the development of protocols and guidelines for ICI treatment with maximized curative potential.
